# Traumatic Ulcerative Granuloma with Stromal Eosinophilia Treated with Intralesional Injections of Triamcinolone Acetonide: A Case Report

**DOI:** 10.3390/reports8040254

**Published:** 2025-12-02

**Authors:** Daniele Pergolini, Angelo Purrazzella, Mohamed Mohsen, Cira Rosaria Tiziana Di Gioia, Antonella Polimeni, Gaspare Palaia

**Affiliations:** 1 Department of Oral and Maxillo-Facial Sciences, Sapienza University of Rome, Via Caserta 6, 00161 Rome, Italy; daniele.pergolini@uniroma1.it (D.P.); mmohsen3010@gmail.com (M.M.); antonella.polimeni@uniroma1.it (A.P.); gaspare.palaia@uniroma1.it (G.P.); 2 Department of Radiological Sciences, Oncology and Pathology, Policlinico Umberto I, “Sapienza” University of Rome, Viale Regina Elena 326, 00161 Rome, Italy; cira.digioia@uniroma1.it

**Keywords:** steroid therapy, intralesional infiltrations, oral pathology, eosinophilic ulcer, traumatic ulcer

## Abstract

**Background and Clinical Significance:** Traumatic ulcerative granuloma with stromal eosinophilia (TUGSE) of the oral cavity is a chronic, rapidly developing mucosal lesion with an unclear pathogenesis, manifesting as a solitary ulcer. Given the malignant clinical appearance of the lesions, it is crucial to ensure the accuracy of the diagnosis to avoid unnecessary invasive surgical interventions. **Case Presentation:** We present a case involving a 69-year-old female affected by a wide, painful ulcer on the left margin of the tongue. An incisional biopsy was performed, and histopathological examination confirmed the diagnosis, revealing a neutrophilic inflammatory infiltrate with components of eosinophils and lymphocytes. Considering the condition’s reactive and inflammatory nature, we planned a corticosteroid treatment with intralesional injections of triamcinolone acetonide. This therapy delivers the active principle directly to the tissues beneath the ulcerative lesion. In three treatment sessions, we achieved the complete regression of the lesion’s signs and symptoms. During a one-year follow-up period, no recurrences were reported. **Conclusions:** The scarcity of documented cases and the ambiguity of definitions in the scientific literature highlight the importance of clinical reports, which refine scientific knowledge about this condition. At the same time, we record an effective and non-invasive treatment that could facilitate healthcare professionals in managing these types of oral pathologies.

## 1. Introduction

Traumatic ulcerative granuloma with stromal eosinophilia (TUGSE) is a chronic, reactive, self-limiting lesion of the oral cavity with an uncertain pathogenesis, which can mimic malignancy due to its clinical similarities to ulcerative squamous cell carcinoma (uSCC) [1]. It presents as a wide, solitary, non-healing ulcer with elevated and indurated borders and a fibrinous ulcer bed [1,2]. The oral areas most commonly involved are the tongue, lips, and buccal mucosa, frequently with a history of repeated trauma, although several cases lack this association. TUGSE differs from common traumatic ulcers because, in the latter, the traumatic aetiology is always identifiable, and healing is achieved by removing the cause [2,3]. In traumatic ulcers, the histopathological examination does not always reveal the deep, peculiar inflammatory infiltration seen in TUGSE biopsy samples [4,5].

Various terms have been used in the scientific literature as synonyms to describe this condition, including eosinophilic ulcer (EU), eosinophilic granuloma of the tongue, and traumatic granulation. The numerosity of synonyms and definitions can be a challenging issue for the clinical management of this pathology, underscoring the importance of reaching a consensus among experts [5,6,7].

Moreover, the TUGSE shows some analogies with the Riga-Fede condition, which is restricted to paediatric patients and is associated with repeated trauma caused by deciduous dentition [8]. In Riga-Fede disease, the most commonly affected oral areas are the apex and the anterior margins of the tongue. The ulcerative lesions are localised in direct association with the position of tooth eruption [8,9].

The histopathology of the TUGSE is characterised by a rich inflammatory infiltrate of neutrophil and eosinophil cells, which are found deep into the connective tissue and the underlying muscle tissues. Another frequent histopathological finding is the infiltration of lymphocytes and plasma cells, which have been shown to be reactive to the CD30 antigen, potentially associated with a spectrum of CD30+ lymphoproliferative disorders [10,11].

In the scientific literature, various treatment options are available to professionals for managing this condition [12]. The wait-and-see strategy, laser photobiomodulation, topical steroid mouthwashes, local steroid infiltrations, systemic steroid therapy, and retinoid medications are described as non-invasive treatment options. Advanced treatment options include cryosurgery and complete surgical excision [13,14].

In this report, we present the case of a patient diagnosed with TUGSE and treated with calibrated intralesional infiltrations of triamcinolone acetonide. The description of diagnostic methods, combined with an analysis of histopathological findings, will help promote an understanding of this pathology and improve clinical management. Moreover, our review of the treatment we propose can serve as a model of an effective, non-invasive therapy, representing a valid alternative to aggressive surgery.

## 2. Materials and Methods

**Figure 1:**
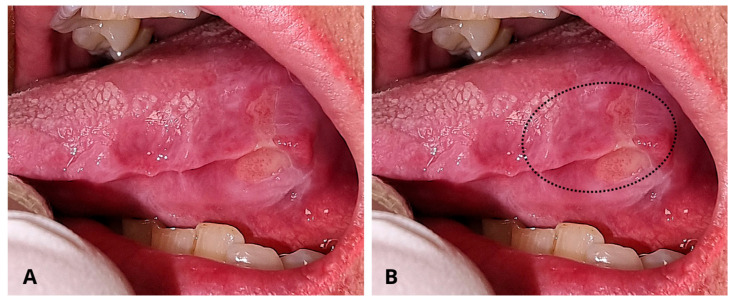
(**A**) Intraoral pre-operative aspect of the ulcer on the left margin of the tongue. The ulcer shows a yellowish central area and indurated margins. The togue margin exposes the indentation of the teeth occlusion. (**B**). The dotted line indicates the area of biopsy.

A 69-year-old Caucasian female was referred to the Department of Oral and Maxillo-Facial Sciences for the clinical evaluation of a wide ulcer on the left posterior margin of the tongue (Figure 1).

The patient had a medical history of erosive oral lichen planus, type 2 diabetes mellitus and stomach neuroendocrine tumour. She had regular prescriptions for the following medications: metformin hydrochloride and cholecalciferol. Moreover, she was under a 6-month follow-up period after systemic corticosteroid therapy was administered for the treatment of oral lichen planus. The OLP lesions were never found on the patient’s tongue, but they presented away on the buccal mucosae. The patient was a non-smoker with no history of smoking or alcoholism.

The intraoral examination revealed a wide ulcer (2 cm × 3 cm) on the left posterior margin of the tongue. The ulcer borders appeared erythematous, elevated and indurated while the central bed showed flat yellowish fibrin. There were no palpable lymph nodes. Due to her multiple chronic health conditions, the patient demonstrated signs of frailty, a condition marked by heightened vulnerability to stressors resulting from diminished physiological reserves.

The patient reported that the lesion first appeared 3 months prior, increasing in size and causing discomfort, and that it did not heal spontaneously. She did not recall any trauma suffered in the oral area where the lesion manifested.

The patient had previously received a topical therapy with a hyaluronic acid and amino acids gel, which yielded no clinical improvement. Also, selective cusp grinding was performed to mitigate the tooth indentation lesions on the tongue margin.

Laboratory investigations, including complete blood count, leukocyte differential, coagulation profile, and glycaemic parameters, were conducted and yielded results with no detectable discrepancies. The panoramic x-ray was examined and confirmed to be clear of any findings.

Given the nonspecific clinical features of this lesion, the differential diagnosis includes infectious diseases, autoimmune diseases, squamous cell carcinoma, drug-related lesions, traumatic lesions, non-Hodgkin’s lymphoma, and TUGSE.

Considering the patient’s medical history and the evocative clinical features resembling malignant lesions, an incisional biopsy was planned after the prescription of blood chemistry and coagulation tests.

The biopsy was performed using a cold blade to obtain a sample comprising the centre of the ulcer and the margins, with a safe margin of supposedly normal mucosa. We chose this site for the biopsy to analyse possible differences between the borders and the ulcer centre, especially regarding the inflammatory infiltrate and the risk of malignant transformation. The surgical specimen was preserved in neutral-buffered formalin solution (10%) and sent to the pathologist for histological evaluation, with a suture knot placed on the anterior margin for orientation.

**Figure 2:**
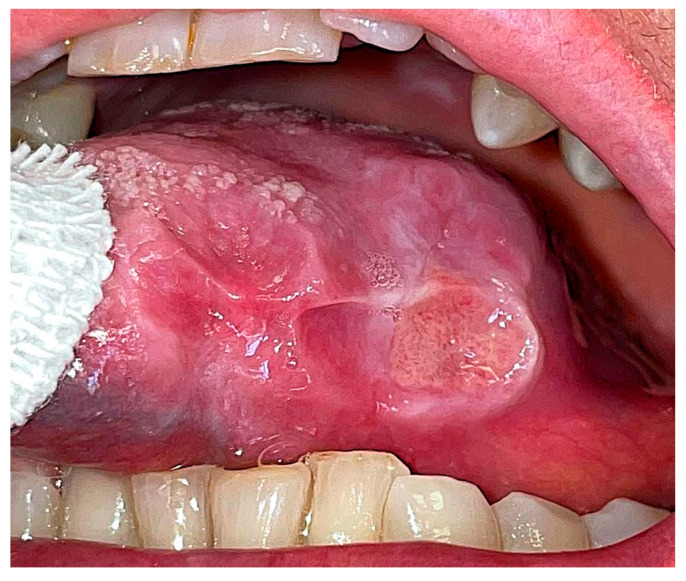
Intraoral post-operative aspect of the ulcer after 1 month from biopsy.

The wound was sutured with five interlocked, continuous, resorbable stitches and healed by primary intention within 3 weeks, without any complications. In the subsequent month, the residual part of the ulcer increased in dimensions (Figure 2).

Gross examination showed that the surgically excised mass measured 1.3 cm × 1 cm × 0.7 cm.

The histopathological examination showed granulation tissue with an infiltration of neutrophil granulocytes and a proportion of eosinophil granulocytes, which can be distinguished by their intense pink cytoplasm and bilobed nuclei. A lymphocyte and plasma cell component was detected, along with blood vessels featuring activated endothelium.

**Figure 3:**
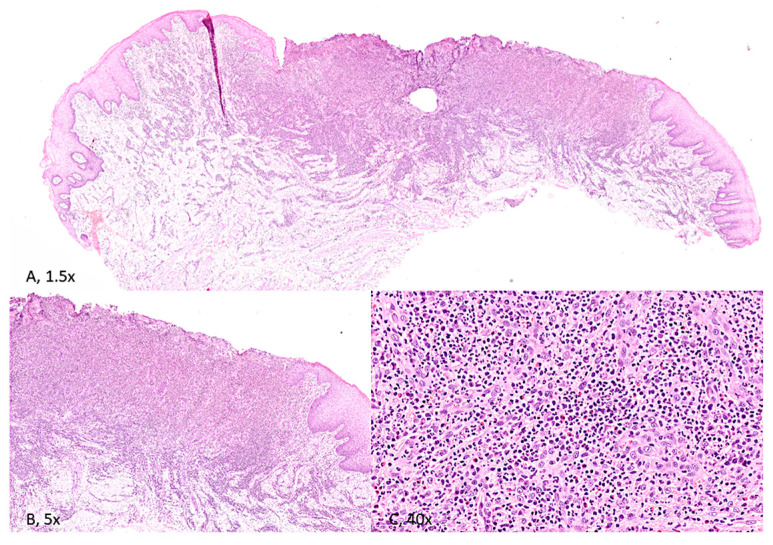
(**A**) [H&E, 1.5× magnification] and (**B**) [H&E; 5× magnification]. Stratified squamous epithelium of the tongue, widely ulcerated with associated inflammation extending deep into skeletal muscle. (**C**) [H&E; 40× magnification]. The inflammatory infiltrate consists of acute and chronic inflammatory cells, with scattered eosinophils.

The inflammatory infiltration was found deep within the connective tissue and the underlying muscle layers, which were partially replaced by fibrous tissue. Ulcer borders revealed low-grade epithelial dysplasia of a metaphlogistic nature, associated with lymphocytes exocytosis figures at the basal layer (Figure 3).

### Case Treatment

The treatment of choice consisted of intralesional injections of triamcinolone acetonide (Kenacort, Bristol Myers Squibb Srl, Anagni, Italy), a corticosteroid in liquid form, to modulate inflammation and promote lesion healing.

For this treatment, we measured the ulcer’s perimeter at each session and administered 0.1 mg of triamcinolone acetonide per square millimetre (0.1 mg/mm^2^).

**Figure 4:**
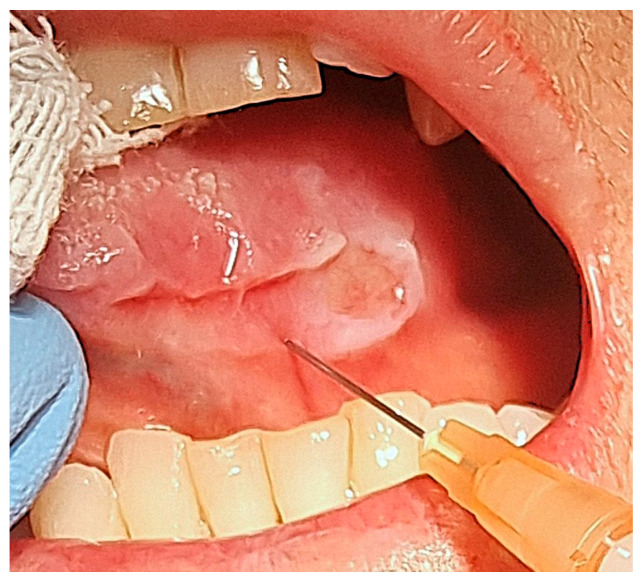
Intralesional injection of triamcinolone acetonide.

After the administration of local anaesthetic without vasoconstrictor, we used sterile insulin syringes with a 30-gauge needle to slowly inject the steroid underneath the margins of the ulcer (Figure 4).

The administrations of the corticosteroid were spaced out by 15 days each. In every session, the patient was reevaluated, and the perimeter dimensions of the ulcer were recalculated to proportion the correct dose of corticosteroid.

As the dimensions of the lesion were regressing, the amount of triamcinolone acetonide injected was recalibrated at each visit based on the calculated diameter.

## 3. Results

**Figure 5:**
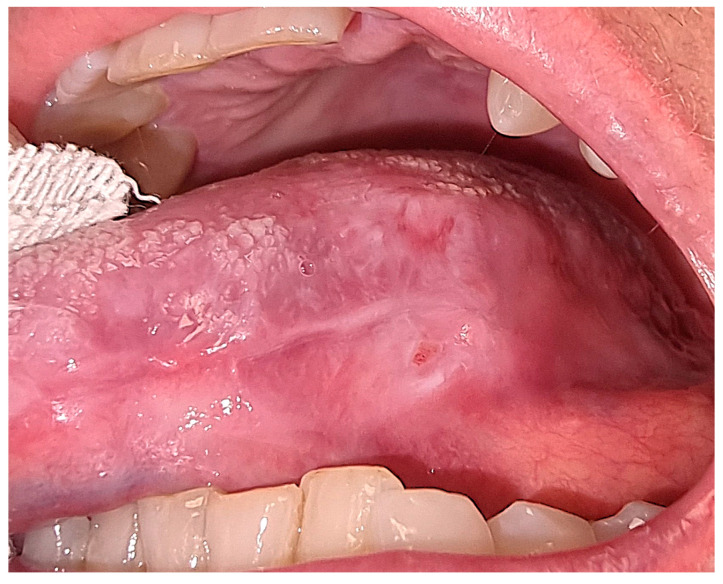
Intraoral aspect of the affected tongue area at the end of treatment, showing the healing.

After three sessions, we achieved complete healing of the lesion and the remission of signs and symptoms (Figure 5).

A maintenance therapy consisting of a chlorhexidine 0.2% mouthwash for oral antiseptic and a hyaluronic acid gel was prescribed, and the patient began the follow-up period. During the follow-up visits, the clinical picture remained stable, and after one year, there were no signs of recurrence of the condition.

## 4. Discussion

TUGSE is an uncommon typology of traumatic lesion which most often occurs on the lateral margins of the tongue but can also affect the lips, buccal mucosa, and the anterior and sublingual tongue mucosa [15]. It has a slightly higher prevalence in female than male patients and is reported more frequently in early childhood (Riga-Fede disease) or after the fifth decade of life. The lesion appears as a broad and deep ulcer with a fibrinous bed and indurated margins [16].

The aetiology and pathophysiology of TUGSE are still unclear. Traumatic events like accidental biting, mobile prosthesis cuts and food injuries are considered to play a fundamental role in the presentation of the lesions. Several cases, however, lack the traumatic aetiology association, underscoring the need for further studies to better understand the characteristics of TUGSE pathogenesis [17].

In the scientific literature, it is also referred to as an eosinophilic ulcer (EU), eosinophilic granuloma of the tongue, or traumatic granulation [18].

The clinical course of TUGSE is chronic, non-autonomously healing and shows discomfort for the patient. In some cases, the clinical appearance of TUGSE can resemble a dysplastic lesion, such as ulcerative squamous cell carcinoma; therefore, a biopsy is recommended to exclude this possibility [17,18].

### 4.1. Histology and Immunohistochemistry

The histopathologic features of TUGSE consist of a deep infiltration of inflammatory cells, such as neutrophilic and eosinophilic cells, which can be found in the connective tissue and the muscle layer beneath the lesion. Often, lymphocytes and plasma cells are found in the inflammatory infiltration. The immunohistochemical examination could reveal the expression of the following antigens: CD3, CD4, CD8, CD20, CD43, CD30, CD68, and vimentin [16]. The eventuality of CD30 reactivity represents a marker associated with a spectrum of CD30+ lymphoproliferative disorders. However, without supportive clinical, histopathological and genetic evidence indicative of a lymphoproliferative disorder, TUGSE should be regarded as a reactive lesion rather than part of the CD30 lymphoproliferative spectrum [16,18].

### 4.2. Differential Diagnosis

Several pathological conditions have similar manifestations to TUGSE; hence a differential diagnosis comprehends traumatic lesions, squamous cell carcinoma, non-Hodgkin’s lymphoma, viral infections, major aphthous ulcers, Wegener’s granulomatosis, histiocytosis X, histoplasmosis, tuberculosis, Langerhans cells histiocytosis, Kimura’s disease, angiolymphoid hyperplasia with eosinophilia, lichenoid lesions, atypical histiocytic granuloma, discoid lupus erythematosus and salivary glands tumours [16,18,19,20]. The diagnosis of TUGSE was based on the histological examination, the patient’s medical history and the clinical features of the lesion. For the latter, we considered the site of occurrence, the negative trauma anamnesis, the unresponsiveness to the removal of possible traumatic factors and the recurrence and aggravation of the lesion after the biopsy.

### 4.3. Treatment

In most cases, TUGSE lesions heal spontaneously after the removal of the traumatic agent that caused them, or healing is frequently reported after biopsy. After the diagnosis, which correlates the clinical and histopathological data, the therapeutic options include a wait-and-see approach, complete surgical excision, topical or systemic corticosteroid therapy, curettage, antibiotics, cryosurgery and laser photobiomodulation [21,22,23,24].

The treatment of choice provided for this case consisted of intralesional injections of triamcinolone acetonide (Kenacort), a corticosteroid. Triamcinolone acetonide is a synthetic corticosteroid widely used for the treatment of oral inflammatory lesions such as oral lichen planus (OLP), graft-versus-host disease, pemphigus, mucous membrane pemphigoid, aphthous stomatitis and oral ulcers [25,26]. It has a potency of 9:1 compared to prednisone, and the local intralesional injection allows for concentrating the immunomodulatory effect specifically under the affected mucosa [27,28]. The choice of intralesional injections over systemic administration avoids the potential side effects that could affect the gastrointestinal system, metabolism, blood pressure, immune system, and sleep rhythm [29,30]. Intralesional injections in this phase permitted avoiding the systemic corticosteroid administration, which would represent an overtreatment considering the frailty of the patient. On the contrary, a topical corticosteroid treatment in the form of gel or mouthwash would not have been sufficiently effective given the difficult accessibility to the area of the lesion. Complete surgical excision was not considered a viable option given the lesion’s benign nature and the importance of the area’s anatomical structures.

Considering the nature of this condition and the importance of the anatomical structures involved, frontline treatment should not include aggressive surgery. Conservative therapy options show excellent results in managing these kinds of lesions, ensuring, at the same time, the comfort of the patient.

## 5. Conclusions

Oral ulcerative lesions are a common manifestation of diverse pathological conditions, and it is essential to deepen scientific research to avoid misdiagnosis. The case of TUGSE reported underlines the importance of a detailed patient diagnosis correlated with a histopathological examination. We successfully treated the patient with a non-invasive and comfortable therapy of local steroid injections. The lesion resolved entirely within a 3-week therapy, requiring only three sessions of administration, thereby ensuring patient comfort. Despite being a benign condition, TUGSE cases require careful follow-up as their nature is associated with lymphoproliferative diseases and autoimmune conditions.

## Data Availability

The original contributions presented in this study are included in the article. Further inquiries can be directed to the corresponding author.

## References

[1] Sah K., Chandra S., Singh A., Singh S. (2017). Eosinophilic ulcer of the tongue masquerading as malignant ulcer: An unexplored distinct pathology. J. Oral Maxillofac. Pathol..

[2] Didona D., Paolino G., Donati M., Didona B., Calvieri S. (2015). Eosinophilic ulcer of the tongue--Case report. An. Bras. Dermatol..

[3] Prabhu Venkatesh D., Ramalingam K., Ramani P., Bhaskaran R. (2024). Traumatic Ulcerative Granuloma With Stromal Eosinophilia (TUGSE): A Case Report. Cureus.

[4] Dhanrajani P., Cropley P.W. (2015). Oral eosinophilic or traumatic ulcer: A case report and brief review. Natl. J. Maxillofac. Surg..

[5] Sidana S.O., Chavan S.R., Baviskar P.S., Natarajan S. (2023). Traumatic ulcerative granuloma with stromal eosinophilia (TUGSE): A rare pathology with unusual behavior. J. Oral Maxillofac. Pathol..

[6] Hannan T.A., Umer M., Syed L., Anis-Alavi M.A. (2020). A case report of traumatic ulcerative granuloma with stromal eosinophilia (TUGSE) in a 21-year-old. Clin. Case Rep..

[7] Bordignon N.C., Correia-Neto I.J., Gondak R., de Albuquerque-Júnior R.L. (2024). Traumatic ulcerative granuloma with stromal eosinophilia mimicking a squamous cell carcinoma. J. Clin. Exp. Dent..

[8] Taghi A., Motamedi M.H. (2009). Riga-Fede disease: A histological study and case report. Indian J. Dent. Res..

[9] SahanaPushpa T., Balamurugan R. (2022). Traumatic ulcerative granuloma with stromal eosinophilia (TUGSE): A rare presentation and case report. Can. J. Dent. Hyg..

[10] Kacar S., Duprez T., Gheysens O., Schmitz S., Van Eeckhout P. (2024). Traumatic ulcerative granuloma with stromal eosinophilia (TUGSE): Case report of a 63-year-old male patient with a rare self-healing oral mucosal lesion. J. Stomatol. Oral Maxillofac. Surg..

[11] Wolk R., Trochesset D. (2025). Traumatic Ulcerative Granuloma with Stromal Eosinophilia: From Reactive Process to Low Grade CD30 + lymphoproliferative Disorder. Head Neck Pathol..

[12] Akhavan A., Mosavi A., Jarrahi M., Navabii H. (2013). Eosinophilic ulcer of the tongue in an 80-year-old Iranian woman after a psychologically stressful event. BMJ Case Rep..

[13] Bortoluzzi M.C., Passador-Santos F., Capella D.L., Manfro G., Nodari RJJr Presta A.A. (2012). Eosinophilic ulcer of oral mucosa: A case report. Ann. Stomatol..

[14] Khaleel Ahmed M., Jafer M., Nayeem M., Hussain Moafa I., Quadri M.F.A., Gopalaiah H., Ali Quadri M.F. (2020). Low-Level Laser Therapy and Topical Medications for Treating Aphthous Ulcers: A Systematic Review. J. Multidiscip. Healthc..

[15] Aizic A., Raiser V., Solar I., Aharon Z., Shlomi B., Kaplan I. (2019). Traumatic Ulcerative Granuloma with Stromal Eosinophilia: CD30 analysis and clonality for T cell receptor gene re-arrangement. Acta Histochem..

[16] Benitez B., Mülli J., Tzankov A., Kunz C. (2019). Traumatic ulcerative granuloma with stromal eosinophilia—Clinical case report, literature review, and differential diagnosis. World J. Surg. Oncol..

[17] Misra S.R., Banerjee A., Das R. (2022). Traumatic ulcer, TUGSE and malignant ulcer on lateral tongue: A trio of similar clinical entities confounding the oral diagnostician!. Oral Oncol..

[18] Fonseca F.P., de Andrade B.A., Coletta R.D., Vargas P.A., Lopes M.A., de Almeida O.P., Santos-Silva A.R. (2013). Clinicopathological and immunohistochemical analysis of 19 cases of oral eosinophilic ulcers. Oral Surg. Oral Med. Oral Pathol. Oral Radiol..

[19] Pergolini D., Mohsen M., Basile F., Marini Grassetti F., Palaia G., Tenore G., Romeo U. (2025). Clinical Correlation Between Antihypercholesterolemic and Antihypertensive Drugs with Oral Lichenoid Lesions: Literature Review and Preliminary Retrospective Analysis. Appl. Sci..

[20] Kachlan M.O., Brooks J.K., Fantozzi P.J., Tenore G., Romeo U., Pergolini D., Sultan A.S. (2025). Oral mucosal adverse events following administration of an immune checkpoint inhibitor: A case report. Gen. Dent..

[21] Del Vecchio A., Tenore G., Luzi M.C., Palaia G., Mohsen A., Pergolini D., Romeo U. (2021). Laser Photobiomodulation (PBM)-A Possible New Frontier for the Treatment of Oral Cancer: A Review of In Vitro and In Vivo Studies. Healthcare.

[22] Falivene G., Pergolini D., Purrazzella A., Todescato L., Mohsen M., Cozzolino P., Palaia G. (2025). Autotrapianto di un canino incluso adiuvato da L-PRF e PBM: Case report. Dent. Cadmos.

[23] Tariq M., Jalli V.V., Dhurubatha J., Medhi R., Malawat K., Gowdar I.M., Arya S. (2024). Clinical Efficacy of Topical Kenacort and Aloe Vera Gel in Minor Aphthous Stomatitis: A Comparative Study. J. Pharm. Bioallied Sci..

[24] Monteiro R.C., Bhat M.R., Martis J., Kamath H.G. (2022). A Comparative Study of the Efficacy of Intralesional 5 Fluorouracil vs Combination of 5 Fluorouracil with Triamcinolone Acetonide in Keloids. Indian J. Dermatol..

[25] Fantozzi P.J., Treister N., Shekar R., Woo S.B., Villa A. (2019). Intralesional triamcinolone acetonide therapy for inflammatory oral ulcers. Oral Surg. Oral Med. Oral Pathol. Oral Radiol..

[26] Xia J., Li C., Hong Y., Yang L., Huang Y., Cheng B. (2006). Short-term clinical evaluation of intralesional triamcinolone acetonide injection for ulcerative oral lichen planus. J. Oral. Pathol. Med..

[27] Qu X., Guo X., Zhu T., Zhang Z., Wang W., Hao Y. (2023). Microneedle patches containing mesoporous polydopamine nanoparticles loaded with triamcinolone acetonide for the treatment of oral mucositis. Front. Bioeng. Biotechnol..

[28] Kuo R.C., Lin H.P., Sun A., Wang Y.P. (2013). Prompt healing of erosive oral lichen planus lesion after combined corticosteroid treatment with locally injected triamcinolone acetonide plus oral prednisolone. J. Formos. Med. Assoc..

[29] Kang K.H., Byun J.S., Jung J.K., Kim J.R. (2025). Mucosal deposit after triamcinolone injection: A case report. BMC Oral Health.

[30] Kuriyama Y., Shimizu A., Toki S., Endo Y., Yasuda M., Motegi S.I., Ishikawa O. (2019). Two cases of chronic oral ulcers effectively treated with systemic corticosteroid therapy: Circumorificial plasmacytosis and traumatic ulcerative granuloma with stromal eosinophilia. J. Dermatol..

